# Asymptomatic Lead Poisoning in a Pediatric Patient

**DOI:** 10.7759/cureus.34940

**Published:** 2023-02-13

**Authors:** Jayani Senanayake, Rangin Haji Rahman, Fady Safwat, Suman Riar, George Ampalloor

**Affiliations:** 1 Medicine, Saint James School of Medicine, Chicago, USA; 2 General Surgery, Saint James School of Medicine, Chicago, USA; 3 Research, Washington University School of Medicine, San Pedro, BLZ; 4 Medicine, John F. Kennedy University of Medicine, Willemstad, CUW; 5 Pediatrics, Humboldt Park Health, Chicago, USA

**Keywords:** atopic dermatitis, blood lead, asymptomatic presentation, pediatric case, blood lead level, seasonal allergies, heavy metal poisoning, heavy metal toxicity, atopic disease, lead toxicity

## Abstract

Lead poisoning is a harmful condition, potentially resulting in irreversible impairments in neurocognition and behavioral development in the pediatric population. Rarely, life-threatening complications may ensue. We report a case of an asymptomatic four-year-old patient presenting with elevated lead levels (74.7 µg/dL) detected on routine blood lead screening at a well-child examination. The patient has a history significant for atopic disease, namely atopic dermatitis, seasonal allergic rhinitis, and food allergies. Overall, the asymptomatic nature of lead poisoning warrants judicious screening in the pediatric population due to the potential for neurologic complications.

## Introduction

Lead is a toxic heavy metal and a common occupational and environmental toxin. Lead exposure may produce serious consequences for the health of children. Asymptomatic lead toxicity has become increasingly common in the pediatric population on account of the element’s ubiquitous presence within the environment and continued use in industry. Children under the age of six years in particular are vulnerable, as it may severely impair their mental and physical development. In 1986, the U.S. Environmental Protection Agency prohibited the use of lead in pipes and solder used for plumbing, though millions of homes are still served by lead service lines [1]. Other risk factors include low socioeconomic status, residing in housing built before 1978, and the use of imported food, medicines, and pottery [2]. Older buildings in particular are common sources of lead poisoning in children due to the presence of lead-based paint and lead-contaminated dust. The old paint will peel off and crumble into dust, which eventually sits on the surfaces inside the home and in the soil surrounding the outside of the home [1]. This poses an issue in children due to their engagement in exploratory hand-to-mouth contact [1]. In addition, adults who are exposed to lead via their occupation or hobbies may inadvertently expose their children second-hand. These occupations include metal welding, shipbuilding and shipbreaking, lead smelting and refining, construction work, and pipefitting and plumbing [1].

Physiologically, lead is involved in the dysregulation of various organ systems, including the hematologic system, renal system, and nervous system. This is largely due to its similarity with divalent cations, namely calcium, zinc, and magnesium [1]. As such, lead is capable of interfering with signaling cascades and mechanisms mediated/regulated by these cations [1]. Hematologically, lead toxicity has been linked to microcytic anemia primarily due to inhibition of delta-aminolevulinic acid dehydratase (d-ALA) and ferrochelatase enzymes, which are essential in the biosynthesis of heme [3]. The cognitive and neurologic effects of lead toxicity in children are thought to be due to the disruption of central nervous system (CNS) development - specifically a disruption in the process of synaptic pruning, which is the process by which extra synaptic connections are eliminated in order to increase the efficiency of pre-existing synapses [3,4]. The cognitive changes are also a result of the inhibition of voltage-gated calcium channels, with lead functioning as an analogue to calcium [3]. Lastly, lead acts at the level of the nephron by impairing proximal tubule function - a site that is responsible for the reabsorption of the majority of filtered solutes [1]. The mechanism by which lead produces its characteristic gastrointestinal findings has yet to be clearly ascertained.

In children, the signs and symptoms of lead poisoning typically present in a non-specific manner, manifesting as headaches, irritability, anorexia, constipation, and abdominal pain [1]. If the dose and duration of lead exposure persist, pediatric patients are at an increased risk of CNS involvement, whether it be in the acute setting or in the chronic, subclinical setting. Acutely, lead encephalopathy presents as clumsiness, agitation, ataxia, seizures, vomiting, and, in severe cases, coma and death [1]. Symptomatic lead poisoning must be treated emergently. Lead encephalopathy occurs in response to lead-induced cerebral microvascular changes, which result in subsequent cerebral edema and increased intracranial pressure. Comparatively, subclinical CNS findings such as cognitive impairment are more common in the pediatric population, and the inverse association between lead exposure and intelligence quotient (IQ) score has been well-established in the literature [1,5].

The diagnosis of lead poisoning is achieved via measurement of venous blood lead levels (BLLs). Current guidelines from the Centers for Disease Control and Prevention (CDC) suggest a BLL at or above 3.5 µg/dL as above normal for a child, based on 97.5 percentile of BLLs among children aged one to five years in the United States [6]. A child with a lead level ≥ 3.5 µg/dL is already considered to be in the top 2.5% of children with the highest lead levels in the United States [6]. Confirmatory testing for children with BLLs greater than or equal to reference range are recommended within one to three months, while BLLs ≥ 45 µg/dL or symptomatic children should be immediately tested within 48 hours [7]. Moreover, additional testing modalities such as complete blood count (CBC) and abdominal and long bone radiographs assist in the diagnosis of lead toxicity. Specifically, CBC indicating reduced hemoglobin and hematocrit levels, along with either normochromic, normocytic anemia or hypochromic, microcytic anemia, as well as venous blood smears with basophilic stippling (representing ribosomal precipitates) are key features of chronic exposure. In the case of acute exposure, abdominal radiographs may demonstrate radiodense lead foreign bodies in the gastrointestinal tract, while long-bone radiographs may be significant for visible lead lines (lines of increased density on metaphysis), signifying growth retardation from chronic exposure [7]. Primary prevention of lead poisoning includes removal of environmental lead hazards from children. Secondary prevention consists of screening tests, namely screening for elevated BLL at routine check-ups [8]. Medical management for children with BLL below 45 µg/dL includes reducing continued lead exposure and frequent BLL measurements, as well as treating any underlying conditions such as iron deficiency anemia and other nutritional deficiencies [7]. Further arrangements to decrease lead exposure should be made through environmental investigation or referrals to lead hazard reduction programs [7]. Chelation therapy and gastrointestinal decontamination using laxatives to remove any ingested lead remain the standard of care for children with a BLL of 45 µg/dL or above. If the potential source of lead exposure has not yet been established or if the patient is living in a non-lead safe house, hospital admission should be considered [7].

## Case presentation

A four-year-old male was evaluated at the clinic for a routine well-child examination, during which he had routine laboratory investigations performed. Laboratory investigations yielded a hemoglobin level of 7.0 g/dL (reference range: 11-13.7 g/dL), hematocrit 28.4% (reference range: 34-44 %), MCV of 47.8 fl (reference range: 75-86 fl), platelet count of 961 K/CMM (reference range: 150- 400 × K/CMM), reticulocytes 3.2% (reference range: 0.5-1.5 %), and elevated BLL of 25.6 µg/dL (reference range: ≤3.5 µg/dL from birth to six years). Other laboratory findings included total iron of 12 mcg/dL (reference range: 60-150 mcg/dL), total iron binding capacity of 460 µg/dL (reference range: 300-360 mcg/dL), transferrin saturation 3% (reference range: 20-50%), and transferrin of 348 mg/dL (reference range: 215-380 mg/dL). A peripheral blood smear was obtained, which revealed a microcytic, hypochromic anemia without basophilic stippling of red blood cells. At the time, the patient was generally well-appearing and asymptomatic. Physical examination was significant for pallor and a new grade one pansystolic murmur heard at all four points. His parents also noted that they had observed him a month prior consuming paint chips at home. It is also worth mentioning that the father’s occupation is in construction, which increases the probability of lead dust exposure from his work environment. The patient consumes an age-appropriate diet and lives with his parents and six siblings. He was started on iron supplementation for his anemia, and a public health inspector determined that their home was found to exhibit elevated lead levels. Repeat laboratory investigations performed a month and a half later revealed a BLL of 74.7 µg/dL and hemoglobin level of 7.1 g/dL, not revealing much improvement since the previous encounter. The patient’s past medical history is significant for atopic dermatitis since one week of birth, food allergies, and seasonal allergic rhinitis. Clinical examination at this visit revealed a well-appearing preschooler with no complaints of abdominal pain, constipation, anorexia, lethargy, irritability, altered mental status, or neurological deficits. Family history is not significant for atopic disease nor lead poisoning in siblings or parents.

The patient was admitted under inpatient care three days following his second outpatient encounter. X-ray of the abdomen and chest completed at the hospital demonstrated no abnormalities apart from slight radio intensities in the right upper quadrant and left lower quadrant and a moderate stool burden. Of note, his physical examination revealed mild abdominal tenderness elicited with palpation, which was thought to be secondary to his plumbism or related to his moderate stool burden. In addition, he also presented with scattered patches of atopic dermatitis spread diffusely across his upper extremities, lower extremities, and the posterior trunk. Notably, the patient had previously experienced moderate pruritus associated with his atopic dermatitis, which had worsened considerably in a two-month span. During his hospital stay, poison control was consulted and the patient was premedicated with diphenhydramine prior to initiating chelation therapy (dimercaprol and succimer), along with a polyethylene glycol laxative to clear fecal matter. His previous medication, ferrous sulfate, was discontinued due to a drug-drug interaction with dimercaprol. On the fifth day of admission, a repeat blood test yielded a rise in ALT level (96 U/L; reference range: 7-55 U/L) and AST (186 U/L; reference range: 8-60 U/L), and eosinophils 10.8% (reference range: 0.0-6%). On the seventh and final day of admission, repeat BLLs were found to be 4.2 µg/dL, and the patient was discharged with succimer by mouth (PO) for 14 days, twice a day. Dimercaprol was discontinued, allowing for the continuation of iron supplementation, and a follow-up appointment was made with his primary care provider to monitor for developmental or neurological abnormalities. The patient’s home was inspected by the department of public health lead inspector who cleared the home for a safe return. Furthermore, the patient’s family and landlord were educated on maintaining a lead-free environment and lead-safe work practices, per the department of health guidelines. There were no complications or new clinical findings identified as of the time of writing.

## Discussion

Lead poisoning is a severe condition whose effects could be recognized across various organ systems. If it remains undetected and untreated, it may result in cognitive impairment and, in severe cases, may culminate with major neurological consequences such as convulsions, coma, and even death in chronically elevated states. Lead poisoning typically results from inhalation or ingestion, with the latter being more common in children on account of their likelihood to ingest inorganic material such as paint chips, ceramic toys, pencil tips, and so forth. Some children have a greater predisposition on account of pre-existent eating disorders or conditions such as pica, as is the case with our patient with a concomitant iron deficiency anemia. In this case, the source of lead exposure was apparent, as the child was observed consuming paint chips, and direct inspection of the home revealed significantly elevated levels of lead. Both of these risk factors highlight the significance of primary prevention. Secondary prevention was also crucial in this case, as it enabled routine lab investigations to be performed and allowed for the early identification and diagnosis of lead poisoning.

From a clinical standpoint, our case of a four-year-old presenting with abnormally elevated BLLs in the setting of an entirely asymptomatic clinical presentation is rather unusual. While the clinical picture in pediatric patients is elusive, the non-specific symptoms that arise are typically dismissed. For instance, irritability may be confused for childhood tantrums, and anorexia may be perceived as picky eating. In addition, constipation may be attributed to lack of dietary fiber, and abdominal pain may be attributed to poor dietary choices. Though lead toxicity is a rare source of abdominal pain, it must be considered in the differential diagnosis of abdominal pain. Our patient did not present with the aforementioned clinical signs. His X-ray imaging did reveal moderate stool burden, which may be directly related to his lead toxicity or an isolated finding altogether as a consequence of poor dietary fiber intake. The patient did present with features of anemia, such as pallor and a new functional systolic murmur; however, his laboratory findings were suggestive of a secondary iron deficiency anemia rather than a primary sideroblastic anemia. Moreover, key findings associated with lead toxicity such as the basophilic stippling of red blood cells on peripheral blood smear and lead lines were noticeably absent from our patient’s laboratory investigations and imaging. Thus, the lack of overt clinical findings, and laboratory findings, apart from the significantly elevated BLL, further reaffirm the ambiguous nature of this case presentation.

Lead toxicity and the clinical sequelae that follow are much more severe in the pediatric population as compared to adults. Physiologically, only 70% (versus 94% in adults) of absorbed lead is deposited in the bones in children, thus leading to a higher BLL [3]. This patient’s lead levels significantly increased within one and a half months. This could possibly be due to his slowed colonic transit time due to a possible lack of fiber in diet as well as his iron deficiency, leading to increased absorption through divalent metal transporter 1 (DMT1). DMT1 is a transmembrane protein and a major iron transporter essential in iron absorption [9]. It is expressed in many tissues of the body, particularly in the small intestine, erythroid cells, lungs, liver, kidneys, and brain [10]. In addition to iron, DMT1 also functions to absorb other divalent cations such as lead and cadmium [10]. Lead shares similar divalent cationic properties with iron, thus resulting in lead behaving as an analogue to iron. This, in turn, leads to altered metabolism of iron and intracellular iron homeostasis [9]. In the setting of iron deficiency, it has been noted that DMT1 increases its absorption and this manifests as lead toxicity. Furthermore, DMT1 exhibits effects of molecular mimicry, as evidenced by inhibition of lead absorption in the presence of iron supplementation in yeast and human fibroblast cells [9].

Another unique laboratory finding in this case was thrombocytosis, with a platelet count of 961 K/CMM (reference range: 150-400 K/CMM). This could be attributed to an acute phase reaction, also termed reactive thrombocytosis. Defined simply, reactive thrombocytosis is an elevated platelet count secondary to infection, inflammation, or hemorrhage [11]. A well-characterized cause of sustained reactive thrombocytosis is iron deficiency anemia, which our patient exhibited [11]. Pathophysiologically, iron deficiency anemia induces a proliferation of megakaryocytes at the level of the bone marrow, thus increasing the platelet count [11]. Another plausible explanation for the thrombocytosis may have to do with the elevated BLL in and of itself. Several studies have reported on thrombocytosis in combination with elevated BLL. This was evidenced in a case study by Al Momen, which outlined a case of a 48-year-old with thrombocytosis in conjunction with elevated BLL from using hair dye every seven to 10 days for seven years [12]. A study published by Katavolos et al. also found elevated platelet counts in trumpeter swans and Canada geese who exhibited elevated BLLs [13]. A study by Shabani et al., which sought to determine the prevalence of abdominal pain in patients who were lead-intoxicated, inadvertently found higher platelet counts in lead-poisoned patients [14]. Contrarily, a study published by Barman et al. found that lead toxicity was associated with low platelet counts in workers exposed to the lead-acid battery plant process [15]. Though the association between platelet count and BLL remains poorly understood, the finding of thrombocytosis in our case supports this association. This association has yet to be analyzed thoroughly in the literature and offers a potential avenue of exploration.

According to the literature, heavy metals have also been linked to worsening symptoms of atopic disease, such as atopic dermatitis [16], seasonal allergic rhinitis [17], and food allergies [18]. Previously conducted research has shown that lead exposure in mice has been associated with enhanced interleukin (IL)-4 production and inhibition of interferon (IFN)- γ leading to activation of Th2-mediated inflammatory response. IFN- γ normally inhibits the proliferation of Th2. Prior studies have found that lead modulates other cytokines such as IL-12 and IL-10, as well as aids in atopic sensitization in humans [19]. Serum IgE levels of children were also noted to be elevated in the setting of environmental exposure to lead [19]. A study conducted on the association of BLLs and sensitization to food allergens in humans concluded that lead exposure did in fact increase the sensitization to food allergens in adults [18]. However, this association has not yet been well established in the pediatric population and requires further investigation. With respect to the laboratory investigations, it was noted that the patient’s eosinophil count was 10.8% (reference range: 0-6%). Although blood eosinophilia is a common finding in atopy, there is something to be said in regard to the association of aggravation of atopic disease in children exposed to high lead levels. While the eosinophilia noted in this patient could support the association between elevated BLL and the aggravation of atopic disease, the baseline eosinophil count could not be obtained, and, as such, the progression of the patient’s atopy, using an elevation from baseline as a surrogate for worsening atopy, could not be ascertained. One finding that necessitates further consideration is the patient’s subjective finding of worsening pruritus. Due to the uncertainty of the patient's onset of climbing BLL, it is difficult to discern whether the worsening of his pruritus is a direct consequence of the elevated BLL or whether it is a natural progression of his atopic disease. A study conducted by Xu et al. found higher lead levels in the epithelial lining of fluid in the nasal mucosa of mice as well as in humans with pre-existing seasonal allergic rhinitis, compared to healthy individuals, when exposed to the same amount of pollen-containing lead [17]. These patients also had worsening symptoms of allergic rhinitis with intranasal exposure to pollen-containing lead in contrast to healthy individuals, possibly due to sticky mucin on the surface of nasal mucosa during activated allergic rhinitis, trapping more lead [17]. These results may corroborate the possibility of exacerbated symptoms of allergic rhinitis in our patient who was also exposed to lead dust second-hand via his father’s occupation. Thus, heavy metal poisoning could possibly be a contributing factor to this patient’s possibly worsening atopic disease. All things considered, these associations have not been well characterized and therefore require further analysis in future research studies.

## Conclusions

Lead poisoning has become increasingly common in the pediatric population and is typically accompanied by vague symptoms such as irritability, abdominal pain, constipation, and anorexia. An entirely asymptomatic presentation, as was seen in this case, is atypical in this patient population. If the dose and chronicity of exposure persist, patients are at a high risk of acute complications (lead encephalopathy) or chronic complications (cognitive impairment). As lead poisoning evidently lacks specific and consistent physical findings, a case-by-case approach to management is necessitated. Overall, this case illustrates the importance of primary and secondary prevention methods of lead poisoning in children, as per CDC guidelines.
